# Supplementary material for: Study of Long-Term Determination Accuracy for REEs in Geological Samples by Inductively Coupled Plasma Quadrupole Mass Spectrometry
Source: Molecules. 2021 Jan 8;26(2):290. doi: 10.3390/molecules26020290 (PMC7827012; doi:10.3390/molecules26020290)
Supplement: Supplementary file 1 [file molecules-26-00290-s001.pdf]

molecules

MDPI

Article

Study of Long-Term Determination Accuracy for REEs in Geological Samples by Inductively Coupled Plasma Quadrupole Mass Spectrometry

Xijuan Tan [1,2,*], Minwu Liu [1,2], Ke He [1,2]

[1] Laboratory of Mineralization and Dynamics, Chang'an University, 126 Yanta Road, Xi'an 710054, China; minwul@chd.edu.cn (M.L.); zyheke@chd.edu.cn (K.H.)
[2] College of Earth Sciences and Land Resources, Chang'an University, 126 Yanta Road, Xi'an 710054, China
* Correspondence: tanxijuan@chd.edu.cn; Tel.: +86-029-8233-9067

Table S1. REEs results of real geological samples with PP bottle as storage container.

REEs		La	Ce	Pr	Nd	Sm	Eu	Gd	Tb	Dy	Ho	Er	Tm	Yb	Lu
S1	1 day	3.87	10.6	1.54	8.72	2.73	1.09	3.59	0.62	4.05	0.87	2.39	0.34	2.15	0.29
	7 days	3.95	10.2	1.52	8.73	2.84	1.08	3.53	0.61	4.26	0.87	2.25	0.33	2.10	0.32
	30 days	3.72	10.1	1.54	8.95	2.72	1.09	3.51	0.59	4.17	0.88	2.26	0.31	2.14	0.28
	6 months	3.86	10.2	1.53	9.03	2.77	1.02	3.46	0.60	4.47	0.86	2.30	0.33	2.23	0.31
	7 months	3.90	10.3	1.55	8.51	2.72	1.05	3.51	0.59	4.10	0.84	2.29	0.30	2.19	0.29
	2σ	0.04	0.07	0.005	0.08	0.02	0.01	0.02	0.005	0.07	0.01	0.02	0.01	0.02	0.01
S2	1 day	2.95	7.83	1.18	6.44	2.17	0.94	2.81	0.54	3.52	0.77	2.28	0.33	2.23	0.34
	7 days	2.96	8.06	1.25	6.62	2.21	1.00	2.92	0.58	3.74	0.84	2.34	0.32	2.29	0.34
	30 days	2.98	7.97	1.16	6.54	1.99	0.99	2.95	0.59	3.65	0.80	2.28	0.31	2.28	0.33
	6 months	3.03	8.23	1.28	6.86	2.11	1.03	2.91	0.57	3.91	0.81	2.40	0.35	2.30	0.32
	7 months	2.96	7.85	1.21	6.66	2.26	0.98	2.91	0.55	3.63	0.78	2.30	0.32	2.24	0.30
	2σ	0.01	0.07	0.02	0.06	0.04	0.01	0.02	0.01	0.06	0.01	0.02	0.01	0.01	0.01
S3	1 day	3.63	9.68	1.49	7.79	2.61	1.00	3.24	0.63	4.03	0.88	2.58	0.36	2.38	0.34
	7 days	3.75	10.4	1.56	7.94	2.61	1.06	3.45	0.68	4.38	0.91	2.61	0.39	2.51	0.32
	30 days	3.37	10.0	1.38	7.51	2.54	0.99	3.36	0.66	3.91	0.88	2.58	0.40	2.39	0.36
	6 months	3.82	10.3	1.55	7.91	2.72	1.04	3.41	0.64	4.23	0.94	2.75	0.40	2.58	0.34
	7 months	3.69	9.81	1.47	7.34	2.49	0.97	3.54	0.65	4.07	0.92	2.62	0.39	2.44	0.35
	2σ	0.07	0.12	0.03	0.11	0.04	0.01	0.04	0.01	0.07	0.01	0.03	0.01	0.03	0.01
S4	1 day	3.63	9.97	1.49	7.74	2.63	0.99	3.30	0.62	3.96	0.84	2.60	0.37	2.36	0.33
	7 days	3.82	10.2	1.59	8.15	2.75	1.01	3.53	0.69	4.13	0.91	2.70	0.37	2.40	0.34
	30 days	3.67	9.58	1.47	7.87	2.73	1.00	3.36	0.68	3.97	0.88	2.57	0.36	2.27	0.35
	6 months	3.84	10.2	1.54	8.40	2.60	1.04	3.50	0.67	4.25	0.91	2.65	0.39	2.53	0.36
	7 months	3.71	9.66	1.55	7.94	2.71	1.06	3.38	0.66	4.05	0.82	2.47	0.37	2.24	0.37
	2σ	0.04	0.12	0.02	0.10	0.03	0.01	0.04	0.01	0.05	0.02	0.03	0.00	0.05	0.01
S5	1 day	3.79	10.1	1.56	8.73	2.75	1.06	3.41	0.66	4.05	0.82	2.47	0.37	2.24	0.37
	7 days	4.00	10.7	1.65	8.71	2.84	1.18	3.53	0.68	4.36	0.93	2.69	0.38	2.42	0.37
	30 days	3.63	9.92	1.57	8.81	2.79	1.10	3.48	0.68	4.46	0.87	2.64	0.36	2.29	0.39
	6 months	3.97	10.8	1.65	8.68	2.80	1.11	3.77	0.69	4.48	0.88	2.83	0.36	2.25	0.40
	7 months	3.78	9.99	1.66	8.79	2.77	1.08	3.70	0.62	4.07	0.86	2.54	0.38	2.33	0.38
	2σ	0.06	0.16	0.02	0.02	0.01	0.02	0.06	0.01	0.08	0.01	0.05	0.01	0.03	0.01
